# Supplementary material for: Are carbon emissions trading and green financial instruments synergistic? -Comprehensive quantitative research based on content analysis
Source: PLoS One. 2024 Mar 7;19(3):e0298601. doi: 10.1371/journal.pone.0298601 (PMC10919601; doi:10.1371/journal.pone.0298601)
Supplement: S1 Data — (ZIP) [file pone.0298601.s001.zip › Data/地级市Z_F工作报告.zip!/░▓╗╒/░▓╟∞╩╨/└┤╫╘╣1⁄2╓┌║┼í╛┬φ┐╦╩2╛▌═°í┐_2018.docx]

——2018年1月4日在安庆市第十七届人民代表大会第一次会议上

市长 陈冰冰

各位代表：

　　现在，我代表市人民政府，向大会报告工作，请予审议，并请市政协委员和其他列席人员提出意见。

    一、过去五年及2017年工作回顾

本届政府任期的五年，是安庆发展进程中极不平凡的五年。在省委、省政府和市委的坚强领导下，我们认真学习贯彻习近平总书记系列重要讲话精神，主动适应和把握经济发展新常态，深入贯彻创新、协调、绿色、开放、共享的发展理念，坚持稳中求进工作总基调，迎难而上，开拓进取，较好落实了“十二五”规划，顺利实施了“十三五”规划，各项工作取得了全面进步。

——综合实力大幅跃升。经济保持较快增长。预计2017年全市生产总值超过1650亿元，年均增长8.7%；财政收入291亿元，年均增长13%，总量居全省第三位；全社会固定资产投资1700亿元，年均增长15.3%；社会消费品零售总额760亿元，年均增长12.6%。科技创新能力不断提高。全社会研发投入年均增长18.1%；建成院士工作站、博士后科研工作站、技能大师工作室等国家和省级研发平台137家，高新技术企业达到193家，发明专利授权量1510件，分别是2012年末的2.6倍、4倍和9.7倍。重大基础设施建设取得突破。宁安高铁开通运营，合安九高铁开工建设，安庆成为国家“八纵八横”高铁网的重要节点城市；岳武高速、望东长江大桥建成通车，实现了县县通高速；国省道里程全省第二；下浒山水库即将下闸蓄水。

——产业结构深度调整。工业经济加快转型。战略性新兴产业产值、高新技术产业增加值年均分别增长13.2%和11%；规模以上工业增加值年均增长10.3%，总量居全省第三位；安庆石化800万吨炼化一体化、江淮新能源汽车、富士康赐福科技研发中心、安庆电厂二期等一批重大项目建成投产。现代服务业健康发展。服务业比重提高了9.1个百分点，电子商务交易额年均增长33%，6个县（市）成为国家级电子商务进农村综合示范县、数量全省第一；主城区建成了新城吾悦、八佰伴、百联等一批城市综合体。农业产业化水平稳步提升。建成“五个百万”基地，农产品加工业产值突破千亿元，稳居全省第二位；省级以上农业产业化龙头企业98家、示范联合体33家，均为全省第一。

——城乡面貌显著变化。中心城市功能日臻完善。累计完成投资450亿元，是前五年的6倍；新改建外环北路、湖心中路等骨干道路89条，改造棚户区750万平方米、老旧小区370万平方米、背街后巷162条；新改建排水管网200多公里，改造易涝点54处；康熙河水系综合治理工程全面完成；新开通城区至海口等公交线路24条，新增营运里程246公里。城镇化建设步伐加快。城镇化率提高了近10个百分点，比全省平均水平快3个百分点；潜山源潭镇等14个镇成为全国重点镇，岳西温泉镇、怀宁石牌镇入选全国特色小镇。农村生产生活条件明显改善。完成农村道路畅通工程7400公里，除险加固小型水库201座，治理中小河流34条，解决了91.4万农村人口饮水安全问题，光纤网络实现村村通；美丽乡村建设水平全省领先，建设省市中心村749个，潜山县成为全国美丽乡村建设综合标准化示范县。

——改革开放持续深化。重点领域改革取得积极进展。“放管服”改革不断深化，市政府部门由39个精简为31个，市级审批项目由357项减少到141项、涉企收费项目由252项减少到50项，新增各类市场主体11.2万户；财税和投融资体制改革深入推进，“营改增”累计为企业减税20.2亿元，累计实现直接融资359亿元、是前五年的6倍，设立了总规模140亿元的产业招商、企业并购等13支基金，8个PPP项目入选全国示范、数量全省第一；农村综合改革取得新进展，土地承包经营权确权登记颁证全面完成，集体资产股份制改革稳步推进。对外开放迈出新步伐。每年一度的北上广招商和台湾经贸活动，扩大了安庆对外影响力；五年累计引进省外资金2750亿元，年均增长24%；安庆（皖西南）保税物流中心（B型）建成运营；安庆港获批全国进境粮食指定口岸和汽车整车进口口岸；安庆海关实现了与全国通关一体化。

——生态文明建设全面加强。主要环境质量指标持续改善。PM10年均浓度由2013年的每立方米113微克下降到77微克、空气质量优良率居全省前列，12个地表水国控考核断面、9个县级以上集中式饮用水源水质均达到考核要求。一批生态建设重点工程推进有力。百万亩森林增长提质工程全面完成，秸秆综合利用提升工程扎实开展，安庆石化新装置区安全卫生防护距离内拆迁工作完成，“三线三边”环境治理和“四治理一提升”专项行动成效明显。绿色产业加快发展。全市旅游业收入年均增长18.5%，风电装机规模突破40万千瓦、光伏发电装机容量超过25万千瓦。

——民生福祉明显改善。脱贫攻坚扎实有力。2014至2016年实现127个贫困村出列、41.4万贫困人口脱贫，2017年126个贫困村出列、14.4万贫困人口脱贫，岳西县将在全省率先摘帽，全市贫困发生率由15.1%下降到3%以下。民生保障水平提高。城乡常住居民人均可支配收入分别达到28600元和11800元以上，是2012年的1.5倍和1.7倍；民生支出占财政支出比重由80%提高到85%；累计新增城镇实名制就业24万人；建成保障性住房6.6万套，改造农村危房16万户；城乡低保标准分别提高了56%和118%。社会事业全面进步。坚持文化惠民，文化“四进”活动年均达到5万场次、服务群众超过1000万人次，圆满举办第七届中国（安庆）黄梅戏艺术节，“黄梅戏·黄金周”影响力不断扩大；义务教育学校标准化建设全面达标，安庆师范学院成功更名为安庆师范大学；医药卫生体制改革深入推进，分级诊疗制度基本建立，医联体、医共体加快建设；市县两级体育场馆建成运营，竞技体育和群众体育活动广泛开展，成功承办省第十三届运动会、第六届残疾人运动会和第七届少数民族运动会。社会大局和谐稳定。安全生产事故起数、死亡人数连年“双下降”，刑事案件立案数连年下降；食品药品安全形势持续向好；社会治理成效明显，人民群众对社会治安的满意度和安全感分别达到92.6%和97.1%；夺取了抗击2016年特大洪涝灾害和灾后恢复重建的重大胜利。

——政府治理能力不断提高。扎实推进法治政府建设，荣获全国“六五”普法先进城市，深入实施“七五”普法，全面实施法律顾问制度，实现重大事项合法性审查和社会稳定风险评估全覆盖。健全完善与市人大、市政协联席会议制度，自觉接受市人大的法律监督和市政协的民主监督，五年来共办理市人大代表议案建议589件、市政协委员提案1782件，办复率均为100%。严格落实中央八项规定精神，持之以恒纠正“四风”，干部作风和精神状态明显好转，队伍素质进一步提高，干群关系进一步密切。切实加强廉政建设，积极推进政务公开，着力营造风清气正的政务环境，五年来共查处侵害群众利益问题944件、处理1365人。注重各项工作统筹，妇女、儿童、老年人、残疾人权益得到保障，民族宗教、外事侨务工作不断加强，国防动员、人民防空、国家安全、地震、气象、涉台、保密、档案、地方志、红十字、关心下一代等工作取得进展。

刚刚过去的2017年，我们大力实施五大发展行动计划，千方百计稳增长、促改革、调结构、惠民生、防风险，预计全市生产总值增长8.5%，财政收入增长8.7%，社会消费品零售总额增长12%以上，城乡常住居民人均可支配收入分别增长8.5%和9%，市十六届人大七次会议确定的各项目标任务均能较好完成。

一年来，我们重点抓了以下工作：

一是着力构建新的产业体系。强力推动各个园区创新发展，聚焦产业首位度，蓄积发展新动能。市筑梦新区在用人、服务、手段等方面形成制度性创新成果，入驻“四新经济”企业近百家。市经开区各项顶层设计加快推进，三期15平方公里基础设施实现“七通一平”，江淮新能源汽车产销量居全国前列，富士康五轴车铣复合加工机批量生产并填补了国内空白，赛富环新电池包项目建成投产，跃迪新能源汽车产业园开工建设。市高新区创建国家级高新区通过初审，北京化工大学安庆研究院挂牌成立，曙光集团搬迁入园项目全面投产。各县（市）区围绕首位产业新上了一批战略性新兴产业项目，集群化发展初具雏形。太湖集友股份成为全国首家通过贫困县IPO绿色通道成功上市的企业，全市新增上市辅导备案企业6家、新三板挂牌企业8家。

二是全面提高城乡建管质量。打造美好大宜城的步伐加快。新改建了皖江大道东段、环城西路、湖心北路等34条道路，完成了“一湖九河”水系治理工程，石塘湖备用水源地建成，破罡湖闸站完工，新增和改造绿化面积70万平方米，“一纵两横”沿路亮化提升工程建成；市立医院东城院区投用，市一中龙山校区、市一院龙山院区、市博物馆加快建设；背街后巷、老旧小区、农贸市场、公共厕所等改造力度加大，城市网格化管理全面推行，城区环卫管理全面提标。新型城镇化水平不断提高。7个县（市）全部进入安徽省文明县（市）先进行列，新建了一批特色小镇和小城镇，出台了非户籍人口在城市落户政策，居住证制度全面实施，城镇化率达到49%。美丽乡村建设深入推进，农村环境“三大革命”扎实开展，72个乡镇政府驻地污水处理设施加快建设，改造农村厕所3万户。

三是更加重视生态环境保护。以共抓长江大保护为导向，切实加强中央、省环保督察和中办专题回访调研反馈问题的整改，按照“基本+”的要求，全面开展环保问题巡查，关停整治小散乱污企业414家，累计查处环境违法行为281起；主城区20蒸吨以下工业燃煤锅炉全面取缔，石油化工等重点行业脱硫脱硝设施全部建成投用；城区污水厂网一体化建设加快推进，谷桥大沟等7个黑臭水体得到整治；全市禁养区内546个规模化畜禽养殖场全部关闭或搬迁；大力实施“河长制”“林长制”，建立了市县乡村四级责任体系。

四是积极创新改革开放局面。完善权责清单、涉企收费清单动态管理机制，建立公共服务清单、行政权力中介服务清单，建成市县乡村一体化的“互联网+政务服务”平台，政府的效能进一步提高；创新城市建设项目招投标方式，推出了10个“工程包”，引进了实力强、信誉好的企业集团，重大基础设施建设的绩效进一步提升；完善金融环境整治联席会议制度，加大涉金融债权案件办理和执结力度，不良贷款率较整治前下降了1.4个百分点，新增贷款超过200亿元，金融服务实体经济的能力进一步增强；出台农村土地“三权分置”办法，农村“三变”改革试点初见成效，潜山县农村产权交易中心挂牌运营，“三农”发展的活力进一步显现；新开通安庆始发直达重庆港的集装箱班轮，安庆港集装箱吞吐量8.2万标箱、同比增长41%，安庆机场航线已增至11条，旅客吞吐量突破45万人次、增长50%，宁安城际铁路的作用日渐放大，去年往返京、沪及沿线的旅客流量突破620万人次，对外开放的基础条件进一步优化。

五是切实加大民生工程力度。把脱贫攻坚作为最大的民生工程。出台了市级层面产业扶贫政策，为全市248个建档立卡贫困村分别注入了20万元的产业引导资金；全市扶贫小额贷款惠及8.1万贫困户，教育扶贫惠及学生11.3万人次，健康扶贫惠及患者21.9万人次；建成183个易地扶贫搬迁集中安置点，安置贫困户10667人；建立104个扶贫驿站，提供了6000多个就业岗位。认真做好公共服务领域的民生工程。市县乡村四级公共文化服务体系建成，义务教育基本均衡县提前两年实现全覆盖，重点人群家庭医生签约服务率达到60%。从民生工程的视角统筹调度公共安全。借鉴巡视工作的办法开展安全生产巡查，一大批关系到人民生命财产安全的隐患得到有效整治；安庆石化危化品码头及油气输送管线迁建工程建成试运行，消除了长期以来威胁城区的最大安全隐患；严格落实信访工作责任制，以解决好群众合法合理合情诉求为重点，信访积案清仓见底、动态清零；全力维护社会稳定，十九大期间维稳安保工作获得省里表扬。

五年来，我们坚持观大势、谋全局、干实事，推出了一系列举措、出台了一系列政策、完善了一系列机制，一任接着一任干，一件事情接着一件事情办，经济社会发展取得历史性成就、发生历史性变革。我们在实践中也积累了很多宝贵的经验，主要是：

（一）拉高工作标杆。我们着力追求更高更快更好发展，坚持“跳起来摘桃子”，很多工作从一开始就瞄准国家级试点、国家级示范，积极争取国家授牌。先后成为国家公共文化服务体系示范区、国家中小学教育质量综合评价改革实验区、国家养老服务业综合改革试点市、国家社会信用体系建设示范市、“宽带中国”示范城市、国家信息消费试点市、中国首个地方戏曲剧种传承发展基地，相继获批国家森林城市、全国国土资源节约集约模范市、全国绿化模范城市，成功创建全国文明城市。人民群众的获得感、归属感、自豪感极大增强。

（二）注重顶层设计。我们秉持先策划、再规划的理念，突出工作系统性，始终谋定而后动。无论是“十三五”规划、调转促行动方案、五大发展行动计划、脱贫攻坚纲要的制定，还是“构建产业新体系、打造美好大宜城”战略的实施，都是面向安庆实际和未来发展，在定向、定位、定线、定调、定盘上下足功夫，同时注重发挥智库的作用，通过政府购买先进服务的方式，先后与安永、毕马威、中和正道、济邦等国际国内顶级咨询机构开展合作，主动吸纳清华大学、浙江大学、同济大学、苏州规划设计院等知名大院大所的研究成果。我们的工作更加理性、更加周密、更有质量、更有效率。

（三）强力推行改革。我们始终致力于创新发展，从规范、管用、绩效的角度做出制度安排，力求人有我用，力争人无我先。一些改革举措已见诸成效：大力精简行政审批事项及流程，率先成为全省“三最”城市之一；以变革园区人事薪酬制度为抓手，着力打造后发地区创新创业高地；不断完善“4+X”政策，对经济社会发展的引导更加精准有力；强力推动要素资源向首位产业集中，产业结构加快转型升级；探索基金招商新路，“四新经济”发展方兴未艾；成功实施了一批PPP项目，政府融资渠道进一步拓宽；建立大安全工作调度机制，社会安全管理全面受控；实行城区基础教育集团化办学，推动教育资源优质均衡配置；积极推行“多规合一”，提升了城市工作的全局性、系统性；建立城市大建设“六分开”、“四到位”制度，城市建管体制更加科学合理、运转高效；推出市政项目“工程包”模式，实现城建工程质量效率双提升；实施环卫运管体制改革，主城区环卫保洁达到“首善要求、首都标准”。锐意改革已成为安庆发展的最强动力和最活源头。

（四）突出开放引领。我们登高望远，始终以更宽眼界、更广胸怀、更大格局推动工作，把抢抓宏观政策机遇、有效整合市场资源作为大策略。在争取国家专项债、棚户区改造、扶贫小额信贷、利用国定贫困县IPO绿色通道、老工业区搬迁改造等政策方面取得了显著成绩；成功引进中远海运、北京环卫、北京城建、北京排水、济南城建、杭州园林等大型央企国企参与安庆的建设与发展；回音必、金田、玉禾田、仁创科技等一批企业总部落户我市。开放发展正给我们不断带来全方位的进步。

（五）主动攻坚克难。我们自我加压，以勇于担当的精神和迎难而上的胆识，解决了许多长期想解决而没有解决的难题，办成了许多过去想办而没有办成的大事。“8828”石化管线迁建、人民路改造、康熙河景观带建设、背街后巷和积水点整治、合安高速互通迁移、石塘湖备用水源地建设、海口通公交等圆满完成，脱贫攻坚、经开区扩区、新能源汽车产业发展、城市框架构建、主城区水系综合治理、合安九高铁建设、铁路道口“平改立”、金融环境整治等取得突破，殡葬改革、森林防火、污染防治、秸秆禁烧等达到预期，历史文化街区连片保护性开发、华阳河蓄滞洪区安全项目、老旧小区改造、城区三轮车整治和烟花爆竹禁限放等推进有力。我们在奋发作为、励精图治中锤炼了队伍、增强了自信。

（六）坚持真抓实干。我们始终坚持干字当头、干就干好、干出一流，做到调度、巡察、督查、考核并重，力求干在实处走在前列。为此，建立了“算着干现场看盯着办”、“两清单一办法”、“日碰头周调度月考核”、“问题导向+解决方案”、“督导式调研”等一系列行之有效的工作机制，形成了“系统谋划、专班跟进、清单管理、闭环运行、高位推动”和“目标导向、行动至上、雷厉风行、快马加鞭”的“安庆工作法”。我们坚信，只要埋头苦干就能创造辉煌。

各位代表！成绩来之不易，经验可资传承。这是省委、省政府和市委坚强领导的结果，是市人大、市政协和社会各界关心支持的结果，是全市人民勠力同心、团结奋斗的结果。在此，我代表市人民政府，向全市广大工人、农民、知识分子、干部、驻宜解放军指战员、武警消防官兵和政法干警，向各民主党派、各人民团体和社会各界人士，向关心支持安庆建设发展的中央和省驻宜单位、海内外友好人士，向在我市创业发展的投资者、建设者，表示崇高的敬意和衷心的感谢！

在肯定成绩的同时，必须清醒地看到，我们的工作还存在许多不足，也面临不少困难和挑战，发展不充分、不平衡的问题仍是主要矛盾。主要表现在：产业结构不优、科技创新能力欠缺、经济外向度较低，发展的质量和效益均亟待改善；中心城市带动能力不强，县域经济实力偏弱，城镇化率仍低于全国全省平均水平；民生领域还有不少短板，脱贫攻坚任务艰巨，城乡居民收入水平总体不高；生态文明建设还需加强，社会文明程度有待提高；政府工作人员适应新形势新任务的能力和水平还不能满足要求。这些问题，我们将努力加以解决。

二、未来五年的奋斗目标和2018年工作安排

今后五年发展的总体目标是：

——经济发展实现更高质量。经济总量保持全省前列，以实体经济为主体、战略性新兴产业为引领、高新技术企业为支撑的现代化产业新体系基本形成，首位产业更具竞争优势，现代服务业成为重要增长点，经济外向度大幅提升，一批企业成功上市。

——城乡发展更加协调。中心城市能级全面提升，县域经济实力明显增强，城镇化率达到全省平均水平，乡村振兴取得重要进展，基础设施网络建设迈上新的台阶。

——生态环境更加友好。大气、水、土壤污染得到有效防治，山水林田湖草系统治理取得明显成效，生态系统保护修复全面推进，绿色产业长足发展，绿色生产普遍推行，绿色生活成为风尚。

——人民群众更有获得感。就业更加充分，优质公共服务更能满足群众需求，社会保障体系更为完善，社会治理体系不断健全，脱贫攻坚任务圆满完成，城乡居民收入逐年提高。

2018年是全面贯彻党的十九大精神的开局之年，也是新一届政府的工作元年，做好今年工作意义重大。

今年政府工作的总体要求是：全面贯彻党的十九大精神，以习近平新时代中国特色社会主义思想为指导，按照省委、省政府的战略决策和市委“456”的总体部署，坚持稳中求进工作总基调，落实高质量发展要求，坚定践行新发展理念，更加注重发展的质量和效益，更加注重发展的协调性和平衡性，更加注重人与自然和谐共生，更加注重深化改革和开放合作，更加注重民生保障和社会治理，坚决打好防范化解重大风险、精准脱贫、污染防治三大攻坚战，为全面建成小康社会、建设现代化新安庆打下坚实基础。

今年经济社会发展的主要预期目标是：全市生产总值增长8.5%以上，财政收入实现同步增长，社会消费品零售总额增长12%，全社会固定资产投资增长11%，城乡常住居民人均可支配收入分别增长8.5%和9%，居民消费价格涨幅控制在3%左右，城镇调查失业率控制在5%以内，脱贫攻坚、污染防治、节能减排、安全生产完成年度目标任务，社会大局保持和谐稳定。

今年的重点工作是：

（一）深入实施“五大发展行动计划”

一是大力推进创新发展，构建现代化产业新体系

深入推进园区体制机制创新、科技创新、手段创新，加快发展战略性新兴产业，培育高新技术企业和上市公司，战略性新兴产业产值增长15%以上，新增高新技术企业70家以上、上市报会企业5家以上。

市经开区着力创新体制机制，积极开展好经省批准的相对集中行政许可权改革试点工作，运用更加灵活的行政审批权和用人自主权，释放园区更大发展活力。新能源汽车产业：依托江汽集团成立合资公司，引入新能源汽车研究院和全产业链配套企业，争取安庆新能源汽车有限公司获得独立生产资质，确保实现年产5万辆的目标；力争跃迪新能源汽车一期建成投产；新能源汽车产业形成百亿规模。高端装备制造产业：在做大做强富士康赐福科技、环新等同时，新引进10家以上企业落户，开工建设北钜智能化数控机床等5个项目，建成智能化无人工厂等5个项目，高端装备制造产业产值达到120亿元。

市高新区实施体制机制创新，加快公共科技服务平台建设，聘请国内顶级科研院所编制产业发展指南，成功创建国家级高新区。医工医药产业：成立股权投资基金和创新人才奖励基金，建成安庆生物医药创新中心、生物医药分析检测等公共平台，抓好华恒生物、鼎旺医药等项目的建设、投产、达产，力争山口片区引进5家以上亿元企业落户。化工新材料产业：积极承接安庆石化百亿元的炼油结构调整及化工新材料项目，新引进具有国际先进水平的项目5个以上，抓好长华氨基甲酸酯、金善三苯基膦等项目的建设、投产、达产，新增化工新材料产业产值20亿元以上。

市筑梦新区巩固改革创新成果，用好招商、孵化等基金，以二期建设为牵引，打造“双创”高地。深度对接各县（市）区首位产业，充分发挥引领、支撑、服务作用，打造现代化产业新体系建设的先行区。按照全过程、全链条、全要素的要求，不断集聚和完善各类创业服务机构，形成全国一流的创新创业生态环境。瞄准“总部经济”、“四新经济”，新引进企业100家以上，力争物色和培育1-2家“独角兽公司”，着力引进1-2家上市公司和2-3家上市后备企业。

各县（市）区按照“一县一区”和“一区多园”的要求，加快资源整合，创新体制机制，建设有竞争力的特色园区和产业基地。桐城市继续深化与央企合作，着力打造全国知名的示范基地；加快形成节能环保和高端装备制造“两个百亿”产业集群；发挥好盛运集团作为上市公司的龙头作用；争取金田新材料公司报会。怀宁县着力深化与中国工程物理研究院成都中心、合肥国家大学科技园等大院大所合作，运用好基金招商等方式，引进新材料和高端装备制造产业项目10个以上，建成液晶面板保护膜等10个项目，两大产业产值分别达到60亿元和80亿元；力争三环集团报备。潜山县以天柱山旅游公司股份制改造为契机，做大做强旅游与养生产业；采取委托北京任科平医药公司开展专业招商等方式，引进医药类亿元项目5个以上，建成奥力欣生物科技等5个项目；力争旅游养生和医药生物产业产值均超过50亿元；争取华业香料公司上市、海南卫康公司报备。岳西县充分利用生态优势和IPO绿色通道，大力开展医药健康和大数据产业的企业招商；发挥好回音必等企业总部落户的聚合作用；谋划好大别山“康谷”“云谷”的建设；争取玉禾田、仁创科技公司上市。太湖县注重放大集友股份的上市效应，加快募投资金项目建设，引进一批关联企业集聚发展；抓好功能膜技术开发、公共实验室、产品检测检验中心等科技服务平台建设，建成富印产业园等8个亿元以上首位产业项目；推进宏宇五洲公司报会、金张科技公司报备。望江县进一步依托现有产业基础，以申洲集团为龙头、童装城为平台建设纺织服装基地，纺织服装产业产值突破150亿元，申洲集团力争成为百亿企业；以安达尔汽车公司为龙头建设新能源客车生产基地，新能源客车产量达到3500辆、产值突破25亿元；推进金泉科技公司报备。宿松县以“两化融合”为抓手，加快优品、欧沃等智能终端项目建设，力争电子信息产业产值突破30亿元；加快以红爱服饰为龙头的纺织服装智能生产基地建设，力争产值超过130亿元；推进森达电器公司报备。迎江区聚焦高端产业，加快都市产业园建设步伐，引进研发设计、文化创意等企业70家以上，生产性服务业产值达到160亿元；聚焦产业高端，依托华茂集团和意大利产业园，循着供给侧结构性改革方向，打造高端纺织产业集群。大观区抢抓老工业区搬迁改造的政策机遇，推动老企业退城进园和升级改造；以飞凯高分子材料等为基础，建成飞凯液晶合成等3个项目，新引进亿元项目5个以上，建设国内有影响力的精细化工产业基地，产值达到80亿元。宜秀区借助北部新城高教资源的优势，积极实施“双创”工程；对现有的园区进行“腾笼换鸟”，聚焦食品工业，建成旺旺智能仓储物流项目，引进一批优质食品加工企业落户，打造百亿食品工业园。

　　二是大力推进协调发展，培植县域经济新优势

坚持扬长补短、稳中求进，紧扣提高新型城镇化水平和乡村振兴，推进城乡协调发展，城镇化率提高2个百分点以上。

注重提升县域经济发展的承载力。立足产业基础、资源禀赋和比较优势，着力打造桐城、望江、宿松县域中心城市，加快建设潜山、岳西、太湖生态特色城市，形成多极支撑的发展格局。桐城市瞄准全国百强县、全省十强县目标，坚持工业立市，重点建设好中建材、金田等多足鼎立的工业园区；坚持商贸活市，加快推进京东智慧物流园、大宗羽绒原料交易市场建设，大力发展现代服务业；坚持强镇发展，突出新渡、双港、孔城等产业特点，建设好一批重点镇和专业镇。怀宁县瞄准全省十强县目标，按照同城化发展的取向，加快与安庆主城区和高铁新站区在基础设施、产业布局、生态建设等方面全面对接；推进工业转型升级，大力发展特色农业；建设石牌、月山、黄墩等一批产业特色鲜明的集镇。潜山县围绕撤县设市，全面提升城市规划建设管理水平；大力发展医药健康产业，充分发掘旅游资源；加快官庄、黄铺、天柱山等特色小城镇建设；整县推进美丽乡村标准化建设；实现在全省争先进位。岳西县在贫困县摘帽的基础上，依托司空山、彩虹谷、明堂山等旅游品牌，推进天堂、河图、店前等旅游集散地建设，建成一批特色酒店、主题民宿、精品农家乐；创建全国有机食品示范基地。太湖县做好旅游强县文章，以创建禅源太湖5A级景区和国家全域旅游示范区为抓手，深化与省出版集团的战略合作，打造寺前等旅游特色小镇，完善各项配套设施，开发旅游衍生产品，提高旅游业对强县富民的贡献率。望江县围绕建设长江经济带绿色产业示范基地，科学利用长江岸线资源，抓紧谋划漳湖、赛口片的产业定位，加快推进桥港现代物流园建设，努力提升经济外向度。宿松县发挥滨临长江和三省交界的地理优势，主动西承武汉、南接九江，用武汉的科教资源，补九江的产业链条，贸易集散辐射周边腹地，打造皖江经济带工贸重镇。

把乡村振兴摆上更加突出的位置。积极构建符合现代农业发展方向的产业体系、生产体系、经营体系、服务体系，加快推进农业农村现代化。深化农村综合改革：落实“三权分置”制度，建设覆盖全市的农村产权交易市场，促进土地资源优化配置；推进农村“三变”改革，加快桐城、怀宁、岳西、宜秀整县试点工作，完成149个试点村改革任务，拓宽村级集体经济和农民的增收渠道。大力发展现代农业：推动一二三产融合发展，培育龙头、引进巨头，打造一批特色优质农产品基地，新增亿元以上产业化龙头企业20家，建设“一村一品”示范村（镇）50个以上；积极培育新型农业经营主体，发展多种形式适度规模经营，新增省级以上示范合作社10家、示范家庭农场20家，培育职业农民5000人以上，耕地流转率达到52%以上；健全农业社会化服务体系，加快培育专业服务公司、专业技术协会、农民经纪人等，新增省级以上服务主体30家以上。改善农村生产生活条件：建设全省“一规四补”示范市，完成下浒山水库工程，做好华阳河蓄滞洪区安全项目、长江安庆河段治理项目前期工作，推进引江济淮工程建设，实施50座小型水库除险加固，治理中小河流9条；深入推进美丽乡村建设和农村环境“三大革命”，全面实施城乡环卫一体化，建设49个乡镇政府驻地污水处理设施，实施10万农村人口饮水安全巩固提升工程，完成农户厕所改造5万个、农村道路畅通工程2000公里。

三是大力推进绿色发展，建设和谐美丽新安庆

以改善环境质量为核心，争创生态文明示范市，促进经济生态化、生态经济化。

积极推行绿色生产和绿色生活。坚定不移发展绿色产业，下定决心实施传统企业绿色化改造，共抓长江大保护。大力发展绿色产业：把全域旅游作为首位的绿色产业，加强全市旅游资源整合，积极创造天柱山景区的上市条件，积极推动潜山、岳西、太湖成功创建国家全域旅游示范区，积极盘活红色之旅、戏曲之旅、禅宗之旅、乡游之旅等题材，旅游业收入增长15%以上；把大健康产业作为优质的绿色产业，推进安庆健康养老示范园、岳西国际养生文化产业园等建设，打造一批分别以温泉、食疗、运动、静修为特色的健康养生基地；把清洁能源产业作为支柱性的绿色产业，新增光伏装机容量5万千瓦，怀宁龙池10万千瓦风电项目建成并网，加快推进望江陈岭风电项目建设，力争桐城抽水蓄能电站开工建设，争取岳西抽水蓄能电站列入国家规划。加快传统企业绿色化改造：落实省工业领域节能环保“五个一百”专项行动，推进华泰林浆纸、永恒动力等30个绿色化改造项目，促进生产过程清洁化、能源利用低碳化、制造工艺绿色化。全面推行绿色生活：大力创建绿色家庭、绿色学校、绿色社区，积极扩大公务用车的新能源汽车配备比例，积极推广新能源公交车和出租车等低碳交通工具，主城区新开通公交线路5条、新增新能源公交车40辆。

坚决打赢污染防治攻坚战。认真落实“大气十条”“水十条”“土十条”，加强污染防治，持续改善环境质量。打赢蓝天保卫战：全面取缔主城区35蒸吨以下和县域10蒸吨以下工业燃煤锅炉，大力整治施工、道路和堆场扬尘，扎实抓好主城区烟花爆竹禁限放，全市秸秆综合利用率达到90%，PM10、PM2.5年均浓度下降比例和空气质量优良率上升比例均高于全省平均水平。加强水污染防治：全面取缔华阳河湖群、沿江湿地省级自然保护区围网养殖，完成新河闸等入河排污口治理，大力整治长江干流安庆段非法码头，所有工业企业实现达标排放，12个地表水国控考核断面水质优良比例达到80%以上，市县两级集中式饮用水源水质均达到或优于Ⅲ类。强化土壤污染管控：推进生活垃圾分类收集，无害化处理率达到100%；开工建设太湖、宿松生活垃圾焚烧发电等项目，建成安庆静脉产业园一期、皖能中科生活垃圾焚烧发电二期工程；开展化肥农药使用量零增长行动，提升畜禽养殖废弃物资源化利用水平。

加强生态系统保护。扎实推进“河长制”，建立“湖长制”，开展河湖清洁行动，推进菜子湖、嬉子湖、潜水河国家湿地公园试点建设。健全完善市县乡村四级“林长制”，推进公路干线绿色长廊、芭茅山改造、青山白化治理等工程建设，修复防护林8万亩、人工造林12万亩以上。开展多要素城市地质调查，实施眉山采石场、独秀山矿区等15个矿山地质环境恢复治理。因地制宜发展涉水、涉林和环保产业，新增油茶种植面积3万亩、蓝莓和茶园各1.5万亩，渔业健康养殖达到10万亩。

四是大力推进开放发展，形成对外开放新格局

以大通道、大平台、大通关、大合作为抓手，发展更高层次的开放型经济，形成内外联动、双向互济的对外开放局面。

建设大通道。推进合安九高铁及沿线站房建设，做好武安杭、随麻安、六安景、宿望宣、安黄铁路前期工作。开工建设岳武高速东延线、德上高速桐城段，启动蕲太高速前期工作，完成安庆至桐城、安庆至望江等5条一级公路建设，加快建设安庆至枞阳、望江至宿松等7条一级公路。推动长江干线航道等级提升，争取8米维护水深常年到沟口段、安庆至武汉航道水深提高到6米的项目启动实施。推进岳西、桐城和天柱山等通用机场前期工作。

提升大平台。努力扩大口岸开放，争取将安庆港长风作业区、皖河农场作业区纳入对外开放范围，并加快推进基础设施建设。在做大做强B保物流中心基础上，积极申创综合保税区，推动保税加工、保税贸易、保税物流全面发展。加快汽车整车进口口岸核心能力建设，建成联合查验、检测、仓储、办公等基础设施，推动与汽车进出口业务相关的产业链建设，争取汽车平行进口试点。发挥粮食进境指定口岸作用，大力发展粮食加工转化产业。加强开放口岸技术保障和应急处置等能力建设，提升口岸运营效率。深入推进通关一体化建设，不断提高通关便利化水平。跨境电商平台建成运营。

开展大合作。坚持引资引技引智相结合，着力引进知名跨国企业，力争外商直接投资增长20%以上。加快建设贸易强市，健全外贸企业孵化培育机制，建设外贸综合服务平台，引进服务外包龙头企业，外贸进出口总额增长10%以上。支持环新、盛运、华茂等企业开展国际产能合作，推动结构调整和转型升级。开展“首位产业招商年”活动，加强与央企、外企、知名民企的战略合作，争取引进亿元以上项目200个以上，其中10亿元以上项目20个以上，新引进项目开工率、到资率分别达到50%和30%以上。

五是大力推进共享发展，满足人民对美好生活新期待

落实以人民为中心的发展思想，以解决人民最关心最直接最现实的利益问题为着力点，进一步增强群众的获得感、幸福感、安全感。

坚决打赢脱贫攻坚战。对照国家考核验收标准，严格落实脱贫攻坚责任制，发挥好“六个专班”作用，强化“单位包村、干部包户”工作考核。继续加大市级财政对脱贫攻坚的支持力度。深入推进“十大工程”，坚持以产业扶贫为根本，将经济组织建在村上，大力推广“四带一自”产业扶贫模式，切实稳定贫困户收入来源、增加贫困村集体经济收入。深入开展“两基建设”提升行动，补齐贫困地区基础设施短板，聚焦贫困地区基本公共服务均衡发展。坚持“扶贫与扶志、扶智相结合”，激发贫困群众的内生动力。确保完成8万以上贫困人口脱贫、100个以上贫困村出列、潜山与宿松2个贫困县摘帽的任务。

提高保障和改善民生水平。突出重点、完善制度、引导预期，在发展中补齐民生短板。促进幼有所育：实施学前教育三期行动计划，普惠性幼儿园比例达到95%，适龄幼儿毛入园率达到86%。促进学有所教：推进义务教育优质均衡发展，实施高中教育普及攻坚计划，高中阶段普及率达到95%；实施特殊教育二期提升计划，残疾儿童少年义务教育入学率达到90%；完善困难学生资助体系，实现建档立卡困难家庭学生资助全覆盖。促进劳有所得：坚持就业优先战略和积极就业政策，千方百计拓展就业岗位，着力解决高校毕业生、农村转移劳动力等重点人群就业，岗前技能培训3万人、岗位技能培训1.5万人、创业培训5000人，新增城镇就业4.6万人以上，农村劳动力转移就业5.6万人，在经济增长的同时实现居民收入同步增长、在劳动生产率提高的同时实现劳动报酬同步提高。促进病有所医：推进健康安庆建设，深化医药卫生体制改革，健全分级诊疗制度，完善医联体建设，医共体实现县域全覆盖，县域内首诊比例达到65%以上；全面落实“351”“180”健康扶贫政策，实现定点医疗机构“一站式”结算和“先诊疗后付费”服务全覆盖；完善统一的城乡居民基本医疗保险和大病保险制度，基本医疗保险参保率100%。促进老有所养：积极推进居家和社区养老服务改革试点，全市167个养老机构服务建设全部达标，每千名老人床位拥有数达到40张以上，低收入老年人服务补贴覆盖率达到50%以上，基本养老保险参保率95%以上。促进住有所居：改造棚户区700万平方米、农村危房1万户，新增商品房500万平方米以上，新建保障性安居工程3.3万套以上，完成公租房分配6000套以上。促进弱有所扶：实行最低生活保障动态管理，做到应保尽保；健全医疗救助、临时救助制度和“救急难”主动发现机制，完善农村五保、生活无着、孤儿及困境儿童等特困人员保障机制；残疾人精准康复服务率达到65%以上；加大对扶老、助残等社会办服务机构的“民办公助”力度。

深入实施文化惠民工程。以文化繁荣为前提，加快地方性优秀文化创造性转化、创新性发展，提升文化影响力吸引力软实力，建设文化强市。支持桐城创建国家历史文化名城。以文化惠民为方向，加强城乡基层文化设施建设，完善市县乡村四级公共文化服务体系，发挥基层文化队伍作用，丰富群众文化体育活动，推进戏曲进校园全覆盖，举办第八届中国（安庆）黄梅戏艺术节，争创国家级黄梅戏文化生态保护实验区，加快建成全国重要的戏剧演出集聚地和戏剧人才培养基地。以文化育人为宗旨，培育和践行社会主义核心价值观，深入实施公民道德建设工程，打响“好人安庆”品牌，巩固全国文明城市创建成果，坚持以市带县、以城促乡，推进全域文明、全面文明。

打造共建共治共享的社会治理格局。加强社会治理制度建设，提高社会治理法治化智能化社会化水平。创新矛盾纠纷多元化解机制：完善重大决策社会稳定风险评估、重大不稳定问题、重大信访事项清单制度，健全行政调解与人民调解、司法调解的联动机制，深化信访工作制度改革，预防和减少社会矛盾。健全公共安全体系：落实安全生产责任制，强化属地管理、部门监管和企业主体责任，加强全社会安全意识和技能培训，完善大安全工作调度机制，持续开展安全生产大巡查，建立风险隐患网格化管控长效机制，坚决防止重特大安全事故，安全事故起数下降5%以上；推进食品药品安全综合治理。完善社会治安防控体系：深入实施“天网工程”“雪亮工程”，提高社会治理预测预警预防能力，提升市县乡村四级综治中心建设水平，推动社会服务管理信息化平台、综治视联网向基层延伸；依法打击和惩治各类刑事犯罪以及黄赌毒黑拐骗等违法犯罪活动，切实增强人民群众安全感。加强基层基础工作：完善村（居）民自治制度，推进民主决策、民主管理，加强村务监督委员会建设，完成村（居）民委员会换届任务；健全农村司法、法律服务、人民调解组织，创建民主法治示范村100个以上；塑造乡村德治秩序，实现政府治理和社会调节、居民自治良性互动。充分凝聚各方力量：发挥工青妇等人民团体的桥梁纽带作用，统筹民族宗教、外事侨务、涉台、国防动员、国家安全、人民防空、地震、气象、保密、科普、档案、地方志、红十字、关心下一代等各方资源，群策群力，共同推进治理体系和治理能力不断迈向现代化。

（二）着力加快区域性中心城市建设

明确以“工贸中心、交通枢纽、开放门户、生活名城”为区域性中心城市的总体定位，使美好大宜城真正成为：经济增长的重要板块、立体交通的重要节点、内陆地区的重要口岸、服务供给的重要所在。

面向未来的奋斗目标是：到2020年，全面提升城市能级，为居民提供更好的教育、更稳定的工作、更满意的收入、更可靠的社会保障、更高水平的医疗卫生服务、更舒适的居住条件、更优美的环境、更丰富的精神文化生活；到2035年，综合经济实力稳居全省第三，主城区经济总量占全市比重达到50%以上，建成一座初具现代化的城市；到2050年，成为长江经济带具有重要影响力的现代化城市。

2018年重点实施“五大工程”：

一是大力实施“畅通工程”。加快交通基础设施建设，构建综合立体交通网络。推进公铁水空互联互通：着手启动安庆北高速互通、海口过江通道的前期工作，认真谋划主城区至高铁新站、高铁新站至新机场的快速通道；完成五里庙港和长风港一期升级改造，启动长风港以集装箱码头为重点的二期建设，开展皖河新港区前期工作；开工建设安庆西高铁枢纽站，规划和预留长风港、皖河新港的铁路专用线；完成天柱山机场航站楼和停机坪改造，推进新机场建设的前期工作。建成城市道路的外环内网：实施沿江东路改扩建工程，打通外环北路与环城西路连接线，开工建设中山大道东段，建成独秀大道三期、天柱山大道东段，完成沿江路西段等道路建设，启动中兴大道高架工程。着力解决停车难问题：按需布局，新建和改造一批老城区停车场。

二是大力实施“双创工程”。强化政策引导，推动实体经济、科技创新、现代金融、人力资源等要素协同，推动互联网、大数据、人工智能等新技术新手段广泛运用。完善科技政策：鼓励产学研深度融合，引进国家级科研院所建立产学研合作平台10家以上，创建省级以上各类创新平台20个以上，实施省级以上科技计划项目不少于30个，全社会研发经费投入增长20%以上，转移转化科技成果100项以上，促进制造业转型升级和现代服务业提质增效。完善金融政策：持续开展金融环境整治，鼓励金融机构创新产品创新服务，重点加强对实体经济尤其是对小微企业的支持，新增贷款230亿元以上；鼓励企业更多地运用直接融资的手段、更好地利用多层次资本市场的资源，直接融资110亿元以上；加快政府融资平台的市场化改造，灵活运用各类引导基金，进一步拉动全社会固定资产投资。

完善人才政策：大力引进行业领军团队和高端人才，吸引市外高层次人才通过柔性引才机制提供智力支持；用好在宜省属和市属高校毕业生资源，留下更多大学生创新创业；加快整合职业教育资源，深化产教融合、校企合作，培养高素质技工大军；鼓励在外安庆籍企业家和各方面人才资智回宜。

三是大力实施“片区工程”。系统谋划城市空间结构，科学编制片区发展规划，采取产业集聚、组团发展的模式，积极培育经济增长点。布局产业新片区：（1）按照主城区副中心的定位，编制完成高铁新站区总体规划，适时启动5平方公里起步区建设，打造知识密集型和高端制造业发展基地。（2）坚持生态优先、环境友好的原则，完善高新区山口片区基础设施建设，在4平方公里起步区内，集聚发展医工医药产业。（3）以经开区三期为载体，聚力打造6平方公里的汽车城，使之成为未来能够容纳50万辆规模新能源汽车生产及配套产业发展的集中区。（4）坚持“多规合一”，着手谋划大观区、高新区、安庆石化协同协作发展的皖河片港城一体化产业新区。（5）统筹集贤关片区与经开区西片开发，完成片区总体规划、土地利用和产业发展规划，引导建设主题公园及关联产业板块。打造特色文化街区：（1）聚焦文商旅融合发展，加快人民路以南历史文化街区保护性开发，基本完成“四街一点”改造，开工建设滨江片区。（2）启动大王庙、大观亭区域的修复工作，打造传统风貌街区。（3）加快东部新城文化地标项目建设，完成市博物馆建设并启动展陈工作，书城综合体项目付诸实施。建设现代商贸物流园区：开工建设海吉星农产品物流园，加快建设皖西南快递物流产业园，谋划建设长风港进口汽车和粮油进出口物流园，规划建设会展中心。

四是大力实施“宜居工程”。坚持以人为本，着力“城市双修”。以棚户区改造为重点推进人居环境建设：推进“两治三改”，坚持“先拆违、后拆迁”，改造棚户区500万平方米、老旧小区180万平方米，完成城区“五化”工程；加强土地供应，坚持多措并举，确保房地产市场健康发展。以河湖连通为重点推进生态体系建设：实施好道桥、涵闸、泵站工程，加快“一纵两横”水系综合整治；推进石塘湖入湖河流治理、老城区河沟生态清淤，实施“四湖两河”截污工程，完成花亭、十里大沟等水体整治；启动秦潭湖、神灵潭、张湖等环境改造；新改建城市绿化200万平方米。以合理分布、优质供给为重点推进公共服务体系建设：规划市立医院东城院区二期项目，打造区域性中心医院；建成市一院龙山院区、市一中龙山校区；“前言后记”书店分别落户市民广场和筑梦新区；抓好国家养老试点城市建设，实施居家养老、社区养老、机构养老+信息化服务平台一体化建设，促进养老与医疗、教育有机衔接，引进专业化、高水准的养老机构，新建居家养老服务站43个，社区养老服务设施配建率达到100%。

五是大力实施“铸安工程”。深化平安安庆建设，织密织牢社会治安、社区管理、安全生产的“防护网”。加强社会治安综合治理：扎实推进全国社会治安综合治理优秀市创建工作，启动大安全大应急联动指挥中心建设，构建大数据实战指挥体系，完善“大情报”和“公安+城管+N”联动联勤机制，建设20个城区公安警务工作站，提高应急处置能力。完善社区治理机制：推动社会治理重心向基层下移，推行社区“微治理”，完善网格化管理机制，发挥公益律师的积极作用，发展志愿服务组织，探索开展面向居民的心理咨询等服务，提升基层治理水平。强化安全生产风险管控：深入开展生产经营活动的安全监管，推进危险化学品安全生产立法，完成安庆石化“8828”油气输送老管线拆除，开展消防安全、特种设备、施工安全以及出租房屋、城中村、城乡结合部等重点领域安全隐患专项治理，确保人民群众生命财产安全。

三、切实加强政府自身建设

决胜全面建成小康社会、开启安庆现代化建设新征程，对政府工作提出了新的更高要求。我们必须始终坚持党的领导，牢固树立“四个意识”，切实增强“四个自信”，以刀刃向内的勇气更新观念，以敢为人先的锐气砥砺前行，以奋发有为的豪气改进作风，努力建设人民满意的服务型政府。

坚持学思践悟，提高施政本领。深入学习习近平新时代中国特色社会主义思想和党的十九大精神，强化理论武装，自觉在思想上政治上行动上同以习近平同志为核心的党中央保持高度一致，坚决维护党中央权威和集中统一领导，坚决忠诚维护看齐习近平总书记这个党的领袖，坚决维护习近平总书记在党中央和全党的核心地位。在全市政府系统营造自觉学习、理性工作、勤于实践、勇于担当的浓厚氛围，倡导“知行合一”和“行知合一”相结合，着力增强学习本领、政治领导本领、改革创新本领、科学发展本领、依法执政本领、群众工作本领、狠抓落实本领、驾驭风险本领，努力造就全体公务人员的“宽肩膀”。

坚持改革创新，优化服务环境。进一步深化“放管服”改革，加大简政放权力度，推进互联网与政务服务深度融合，不断提高政府效能，让便民利民和“老百姓办事不跑第二趟”成为现实。进一步深化商事制度改革，全面实行“多证合一、一照一码”“证照分离”，建立完善商事登记信息共享制度，强化事中事后监管。进一步创新政府绩效管理，加强刚性约束，建立社会评价机制，扩大社会参与，引入第三方评估，推动工作落实。进一步完善清单管理制度，加强权力运行监管，厘清权力与责任关系，深入推进政务公开。

坚持依法行政，建设法治政府。健全依法决策机制，确保程序正当、过程公开、责任明确，全面推行政府机关重大决策合法性审查，注重政府法律顾问队伍建设，加强规范性文件监督管理，提高政府决策的公信力和执行力。坚持严格规范公正文明执法，明确行政执法操作流程，推行行政执法公示制度，加强行政执法信息化建设，强化行政执法人员管理，落实行政执法责任制和责任追究机制，提高行政执法社会满意度。健全行政权力运行监督体系，自觉接受党内监督、人大监督、民主监督、监察监督、司法监督、行政监督、审计监督、社会监督、舆论监督。全面强化政府工作人员的法治素养和法治能力，恪守合法行政、合理行政、程序正当、高效便民、诚实守信、权责统一等依法行政的基本要求，做尊法学法守法用法的模范，在法治轨道上全面推进政府工作。

坚持求真务实，切实改进作风。更加牢记使命、不忘初心，坚持人民至上、以人为本，密切联系群众，增强群众观念和群众感情，凡群众反映强烈的问题都严肃认真对待，凡损害群众利益的行为都坚决纠正。更加用力纠正“四风”，严格落实中央八项规定精神，坚决反对形式主义、官僚主义，重点解决好“表态多调门高、行动少落实差”的问题，领导干部带头抓作风建设，身体力行，以上率下，形成“头雁效应”。更加坚持政贵有恒，以“问题导向+解决方案”的工作姿态，问需于百姓、问计于基层、问效于一线，用真功、谋实招、持韧劲、有定力，靠钉钉子精神把各项工作做实做细做好。

坚持从严治政，推进廉洁建设。认真落实党风廉政建设主体责任和“一岗双责”，强化政治纪律和组织纪律，严肃廉洁纪律、群众纪律、工作纪律、生活纪律，使政府工作人员知敬畏、存戒惧、守底线，习惯在受监督和被约束的环境中工作和生活。切实加强执纪问责，重点关注脱贫攻坚、安全生产、建筑市场、环境保护等领域，坚决查处发生在群众身边的贪污腐败和失职渎职行为。不断加强制度建设，健全廉政风险防控机制，强化不敢腐的震慑，扎牢不能腐的笼子，增强不想腐的自觉，营造风清气正的政务环境。

各位代表！九层之台，起于累土；千里之行，始于足下。让我们紧密团结在以习近平同志为核心的党中央周围，在省委、省政府和市委的坚强领导下，以更加昂扬的姿态，积极投身“从全面建成小康社会到基本实现现代化，再到全面建成社会主义现代化强国”的新征程，奋力谱写“安庆篇章”！
